# Correlation of volumetric flow rate and skin blood flow with cold intolerance in digital replantation

**DOI:** 10.1097/MD.0000000000009477

**Published:** 2017-12-22

**Authors:** Gang Zhao, Jingyi Mi, Yongjun Rui, Xiaoyun Pan, Qun Yao, Yang Qiu

**Affiliations:** a Department of Medicine, Soochow University, Suzhou; b Department of Hand Surgery, Wuxi Ninth People's Hospital, Wuxi, Jiangsu, China.

**Keywords:** cold intolerance, digital replantation, skin blood flow, volumetric flow rate

## Abstract

Cold intolerance is a common complication of digital replantation. The exact etiology is unclear, but it is considered to be multifactorial, including nonsurgical characteristics, vascular, and neurologic conditions. Blood flow may play a significant role in cold intolerance. This study was designed to evaluate the correlation of digital blood flow, including volumetric flow rate (VFR) and skin blood flow (SkBF), with cold intolerance in replanted fingers.

A retrospective study was conducted among patients who underwent digital replantation between 2010 and 2013. Patients were selected into study cohort based on the inclusion criteria. Surgical data was collected on each patient, including age, sex, injury mechanism, amputation level, ischemia time, number of arteries repaired, and whether or not vascular crisis occurred. Patients were included as study cohort with both nerves repaired and without chronic disease. Cold intolerance was defined as a Cold Intolerance Symptom Severity (CISS) score over 30. The arterial flow velocity and caliber were measured by Color Doppler Ultrasound and the digital VFR was calculated. The SkBF was measured by Laser Speckle Imager. Both VFR and SkBF were calculated as a percentage of the contralateral fingers. Comparative study of surgical data and blood flow was performed between the patient with and without cold intolerance. Correlation between VFR and SkBF was also analyzed.

A total of 93 patients met inclusion criteria for the study. Approximately, 42 patients were identified as having cold intolerance. Fingers that survived vascular crisis had a higher incidence of cold intolerance with a lower VFR and SkBF. The VFR was higher in 2-artery replantation, but the SkBF and incidence of cold intolerance did not differ significantly. No differences were found in age, sex, injury mechanism, amputation level, or ischemia time. Furthermore, no correlation was found between VFR and SkBF.

Cold intolerance of digital replantation is associated with decreased SkBF and VFR in the replanted fingers, which survived vascular crisis. Further work will be focused on how vascular crisis cause the decreasing of SkBF and VFR and the increasing chance of cold intolerance.

## 1 Introduction

In the modern medical society, developed microsurgical techniques ensure a high survival rate when performing digital replantation. However, only a patient's functional recovery is a true success. One of the most common complications of digital replantation is cold intolerance, which has been reported as high as 100%.^[1]^ It not only presents as various uncomfortable symptoms, such as pain, numbness, swelling, and skin color change; but also may affect the function of the replanted finger.^[2,3]^ The severity of cold intolerance and hand dysfunction were always evaluated using Cold Intolerance Symptom Severity (CISS) questionnaire, in which the effect on daily life and job were included.^[4]^

Although cold intolerance is ubiquitous, its etiology is poorly understood. It is often considered to be the result of multiple factors, including blood circulation, nerve condition, or injury characteristics. Among them, alteration of blood supply is the common concern, which has been previously reported controversially. Tark et al^[5]^ found that replanted fingers with only 1-artery anastomosis suffered more from cold intolerance. Walaszek et al^[6]^ used ultrasound to demonstrate blood flow resistance in anastomosed arteries of replanted fingers with moderate cold intolerance. These studies indirectly demonstrated the correlation between cold intolerance and the decreased blood supply of replanted finger, but the negative results of the impact to cold intolerance were also reported. Freedlander's^[7]^ study showed that 52% of replanted fingers had a significantly lower skin blood flow with cold exposure than normal fingers, but this difference did not correlate with cold intolerance. Lannau et al^[8]^ also reported no difference in cold intolerance between the arterial patency and occlusion in upper limbs. The reason behind the conflict results was because of the limitation that they did not quantitatively analyze the blood supply of replanted fingers, which could be explained by the volumetric flow rate (VFR) and skin blood flow (SkBF). The VFR is determined by vessel caliber and blood flow velocity, and the SkBF is related to perfusion of skin capillary. However, there are a few reports that evaluate VFR and SkBF in replanted digits and their correlation with cold intolerance. Therefore, it is hard to clearly understand the blood circulation of replanted finger and clarify the effect on cold intolerance.

The current retrospective study was designed to quantitatively analyze the VFR and SkBF of replanted fingers and evaluate their impact on cold intolerance to clarify the relationship between digital blood supply and cold intolerance.

## 2 Materials and methods

### 2.1 Study population

The study was approved by the Institution Review Board of Wuxi Ninth People's Hospital before data collection and analysis. To make sure the minimal follow-up was more than 3 years, the cohort selection was defined from 2010 to 2013. We included patients who underwent finger replantation as the study sample with the following criteria: age at surgery between 20 to 50 years, single finger complete amputation (second to fifth finger), amputation level at proximal and middle phalanx, no other surgery combined, and both digital nerves were repaired. The medical record of each patient was carefully reviewed to collect surgical data including age, sex, injury mechanism, amputation level, ischemia time, number of repaired arteries, and whether postoperative vascular crisis occurred.

### 2.2 Evaluation of cold intolerance

Cold intolerance of the injured hand was evaluated using the CISS questionnaire, which has been widely applied in previous reports. The first part of the questionnaire was not scored, which was composed of 7 questions assessing symptoms including pain, numbness, stiffness, weakness, aching, swelling, and skin color change. The total score of the other parts of the questionnaire is from 0 to 100; a higher score means worse cold sensitivity. Based on study by Ruijs et al^[9]^, cold intolerance was defined as a score over 30.

### 2.3 Measurement of VFR

Hemodynamic study of digital arteries was performed in both the replanted and contralateral fingers by Color Doppler Ultrasound (LOGIQ E9, GE) with a high-frequency mini transducer probe (4–15 MHz, 25 mm footprint, L8-18i, GE). After checking the patency of the anastomosis, the mean flow velocity and inner diameter of the arteries was measured at the level of the distal interphalangeal joint, which was distal to the amputation level. To improve the measurement's accuracy, the angle between the ultrasound beam and the flow direction was adjusted to be less than 60 degrees during examination. The VFR was roughly evaluated using the formula: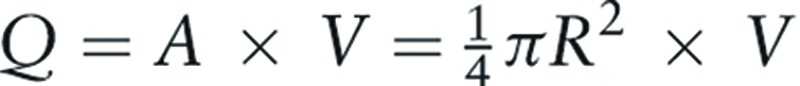, (Q: flow volume in unit time, A: cross-sectional area, V: flow velocity, and R: inner diameter), which is widely used to calculate the pipe flow rate. The total VFR of a finger was calculated by adding the VFR of all feeding digital arteries. To avoid an error caused by differences between separate fingers or individuals, the VFR of replanted fingers was assessed and expressed as a percentage of the result of contralateral fingers according to the following formula: 
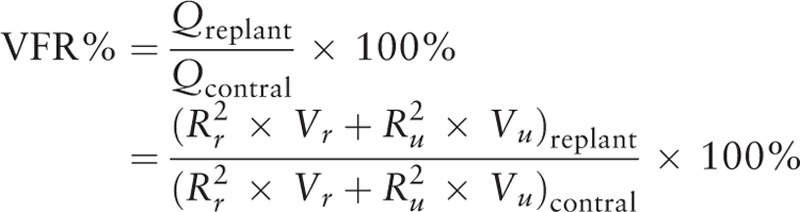


(*R*_*r*_ and *V*_*r*_: inner diameter and flow velocity of radial digital artery, *R*_*u*_ and *V*_*u*_: inner diameter and flow velocity of ulnar digital artery). The VBF of an unrepaired digital artery was considered as zero during calculation.

### 2.4 Measurement of SkBF

After the VFR study, SkBF was measured at the distal pulp of both replanted and contralateral fingers by Laser Speckle Imager (PeriCam PSI System, Perimed, Sweden), which is a novel way to assess skin microvascular function in real time. Patients were required to avoid vigorous exercise, alcohol, coffee, and tea within 2 hours before examination. The room temperature was controlled at 22 to 23°C. SkBF measurement was performed after the patients had rested for at least 20 minutes. Parameters of examination were set as follows: height of the camera was 25 cm, sampling area and duration was 15 cm × 15 cm and 5 minutes, image acquisition rate was 10 Hz, region of interest (ROI) was located on the corresponding pulp of both fingers using the 5-mm circle. Data were collected and analyzed with the software package from the manufacturer. The results were also expressed as a percentage of the value of contralateral fingers to avoid individual differences.

### 2.5 Statistical analysis

All the data was statistically analyzed with the software SPSS 21.0. The means and standard deviations were calculated for the continuous variables, and the frequency was calculated for the categorical variables. Correlated factors were compared between the patients with and without cold intolerance using *t* test or *χ*^*2*^ test according to the type of variables. Further comparative study of the SkBF and VFR was performed between 1-artery and 2-artery replantation, and between the replanted fingers with and without vascular crisis. Finally, we conducted a Pearson correlation analysis between SkBF and VFR in the whole cohort to determine their relationship. Statistical significance was defined as the *P* ≤ .05.

## 3 Results

### 3.1 Difference of general characteristics

Between 2010 and 2013, a total of 141 patients were initially selected. Twenty-one patients were excluded because of failed replantation or chronic diseases, such as hypertension, diabetes, and any other vascular occlusive disease; 27 patients were lost to follow-up. Finally, 93 remaining patients were included in the study who were required to complete the CISS questionnaire and accepted the examination of digital blood flow (Table 1). Forty-two patients (45.2%) suffered cold intolerance after digital replantation. Between patients with and without cold intolerance, there were no significant differences in mean age, sex, injury mechanism (sharp cutting, electric saw cutting, crash injury, avulsion injury), amputation level (middle and proximal phalanx) and ischemia time (<2 h, 2–4 h, >4 h) (*P* > .05).

**Table 1.**
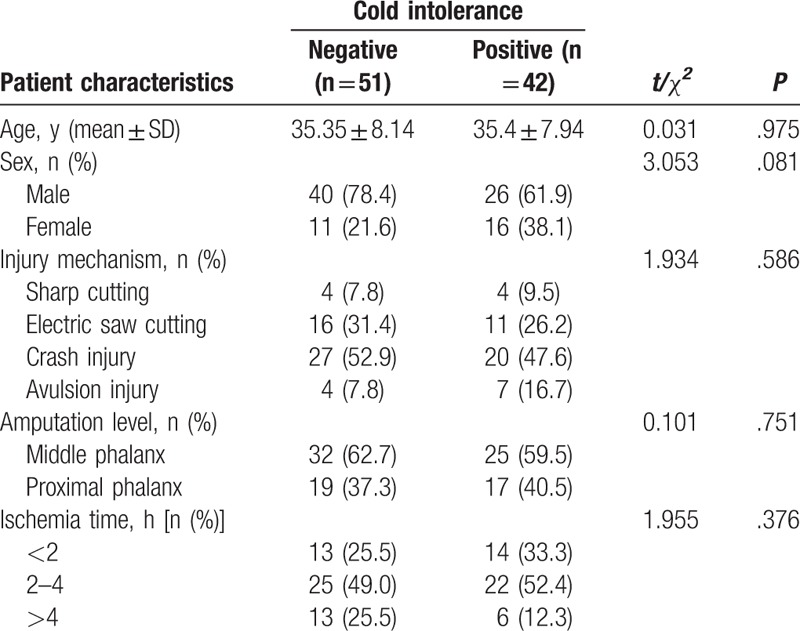
The comparison of patient characteristics between negative and positive cold intolerance groups.

### 3.2 VFR and SkBF in different groups

Based on the medical records of patients, 54 fingers (58.1%) had 1-artery anastomosis and 39 fingers (41.9%) had 2-artery anastomosis. Color Doppler ultrasound found 7 occlusive arteries in 7 two-artery replanted fingers, which meant a patency rate of 94.7% in 132 repaired arteries. Therefore, the number of 1-artery replanted fingers was adjusted to 61 (65.6%) and number of 2-artery replanted fingers was adjusted to 32 (34.4%). The incidence of cold intolerance had no statistical difference between the 1-artery and 2-artery groups (*P* > .05). The VFR was higher in the 2-artery group (*P* < .05), but the SkBF had no difference between the 2 groups (*P* > .05) (Table 2).

**Table 2.**
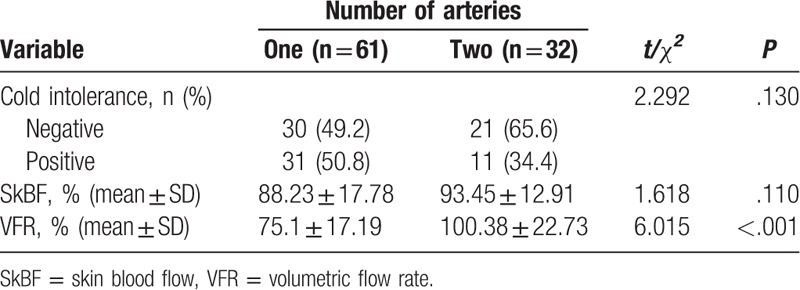
The comparison of cold intolerance,SkBF and VFR between one- and two- artery groups.

**Figure 1:**
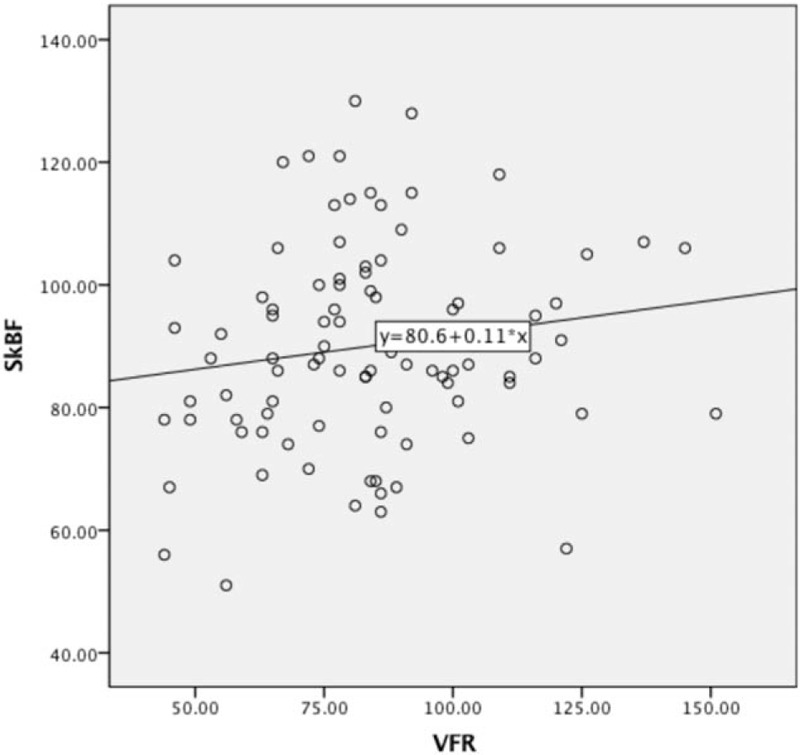
Pearson's correlation analysis showed no correlation between SkBF and VFR.

Comparative study between patients with and without cold intolerance found no significant differences in SkBF and VFR (*P* > .05). On the other hand, Pearson correlation analysis for SkBF and VFR in the study cohort showed no significant correlation (r = 0.157, *P* = .133) (Table 3). A scatterplot is shown in Figure 1.

**Table 3.**
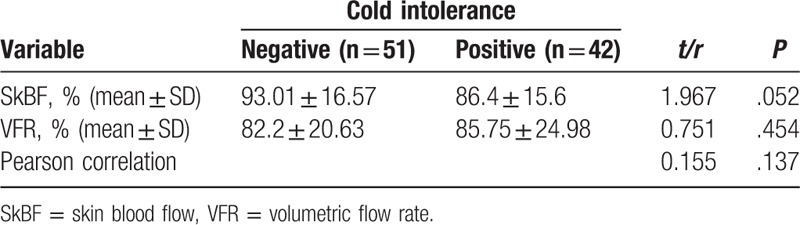
The comparison of SkBF and VFR between negative and positive cold intolerance groups and the correlation between SkBF and VFR.

### 3.3 Cold intolerance of fingers with vascular crisis

In the study, 12 replanted fingers (12.9%) experienced postoperative vascular crisis. Nine (75.0%) of these patients were diagnosed with cold intolerance, which was a higher incidence than fingers without vascular crisis (40.7%) (*P* < .05). Further comparative study showed that both SkBF and VFR were lower in this group (*P* < .05) (Table 4).

**Table 4.**
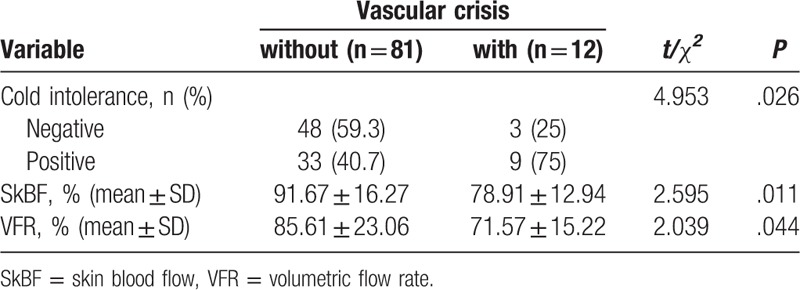
The comparison of cold intolerance,SkBF and VFR between with and without vascular crisis groups.

## 4 Discussion

Cold intolerance is one of the most notable postoperative complications of digital replantation. It is characterized by variable abnormal symptoms with cold exposure, including pain, dysesthesias, stiffness, skin color change, and dysfunction of the hand for work and leisure activities.^[10]^ Cold intolerance has typically and extensively been evaluated with the CISS questionnaire, which has proven reliable with scores ranging from 0 to 100 according to the severity of symptoms.^[11]^ In consideration that cold intolerance can more or less exist in normal people, Ruijs et al^[12]^ conducted a prospective study in healthy volunteers, which suggested that a CISS-score of 30 was the threshold for pathological cold intolerance. In the current study, patients with a CISS score > 30 were defined as having cold intolerance and based on their score were divided into 2 groups: positive and negative cold intolerance.

Although postoperative cold intolerance has been recognized and studied over a few decades, the etiology is still generally unclear. It is considered the result of multiple factors, including patient characteristics, injury mechanism, blood circulation, nerve condition and disturbed thermoregulation.^[13–18]^ As a common concern, the impact of blood circulation to cold intolerance has been previously reported. These studies mainly looked at the number of feeding arteries, arterial patency, flow resistance, and SkBF and their correlation with cold intolerance.^[5,6,7,16,19]^ However, there is no report about VFR of arteries in the replanted finger. In the current study, we calculated total VFR of all feeding arteries to evaluate the blood supply of the finger, and analyzed its relationship with SkBF and cold intolerance.

Similar to most studies, demographic factors including sex and age were not related to cold intolerance in current study. However, a few authors have reported a positive relationship in other studies. Novak et al^[20]^ reported that women had higher CISS-scores with brachial plexus injuries. Sun et al^[21]^ found that younger patients were less likely to have cold intolerance after a digital artery flap. Despite different findings shown in both studies, the objects of those studies were not digital replantation. It is possible that the susceptibility to cold intolerance varies with the type of injury or surgery. Therefore, injury mechanism is assumed as another factor correlated with cold intolerance. It is classified into 4 types: sharp cutting, electric saw cutting, crash injury, or avulsion injury according to the increasing severity of the potential injury to the neurovascular bundle. Although, similar to Craigen et al's^[14]^ study, the result was negative, the influence of injury mechanism can’t be completely ruled out because some patients with extremely serious injury were excluded because of failed replantation. As for the injury level, de Medinaceli et al^[22]^ showed that cold sensitivity was less marked at a more proximal level because of a larger nerve caliber. However, this finding was not reproduced in our study. A possible interpretation is that there is no significant discrepancy of nerve size at the proximal and middle phalanx levels.

The hemodynamic study showed that replanted fingers supplied by 2 arteries had a higher total VFR than those supplied by only 1 artery. However, there was no difference in SkBF between them. Likewise, no significant correlation between total VFR and SkBF was found in the entire cohort, which is consistent with published studies.^[6,16]^ This is not a surprising result because the SkBF is commonly considered to be related to the skin vasomotor reflex (SkVR) responses, which are dominated by sympathetic nerves.^[23,24]^ Furthermore, we failed to find a decreased incidence of cold intolerance in 2-artery replanted fingers, even though they had a higher VFR. However, this is not to say that both arteries should not be repaired when possible during surgery, which may improve the survival rate of the digit.^[25]^ This is supported by our study, where 7 replanted fingers contained arterial occlusion.

Our data showed that those fingers that survived the postoperative vascular crisis, no matter if they underwent surgical vascular repair or nonsurgical salvage, had an increased risk of cold intolerance. This finding may be related to insufficient blood supply, which is reflected by a lower VFR and SkBF compared with those fingers without vascular crisis. The Doppler data demonstrated that the lower VFR resulted from smaller arterial caliber and slower blood flow. We attributed this difference to persistent vasospasm, which has widely been reported in cold environments.^[1,15,19,26,27]^ Noteworthy, the correlation between cold intolerance and lower VFR and SkBF was only found in those fingers with vascular crisis. Therefore, we can only draw the conclusion that vascular crisis has some effect on cold intolerance in digital replantation with lower VFR and SkBF. To explore the reason how vascular crisis correlate with the decreased SkBF and VFR in digital replantation, further work will be performed on it.

There are 2 limitations of the current study. First, because this study was only designed to analyze the effect of blood supply, the number of repaired nerves was unified into neurorrhaphy of both nerves by inclusion criteria. However, the nerve condition is also an influencing factor for cold intolerance in digital replantation. A study for upper-extremity peripheral nerve injuries conducted by Collins et al’ s^[17]^ reported that 38 of 50 patients complained of cold sensitivity, 33 of whom were rated moderate or severe. Novak et al^[20]^ also reported that patients with peripheral nerve injury were more likely to have a high level cold intolerance. Second, the VFR and SkBF were only measured at room temperature. It was reported that cold intolerance of a replanted finger is related to cold-induced vasospasm.^[27]^ Furthermore, this pathologic vasospasm has been found in amputation stumps and normal fingers, not just in replanted fingers.^[1,15]^ Unexpectedly, we found that vasospasm occurred at room temperature as well, which can be inferred from the lower VFR of fingers that survived vascular crisis caused by smaller arterial caliber and decreased flow velocity.

The current study showed that the replanted fingers which survived vascular crisis had a higher incidence of cold intolerance, which was correlated with its decreased SkBF and VFR, but this relationship did not exist in common digital replantation. Therefor, further work will be focused on how vascular crisis cause the decreasing of SkBF and VFR and the increasing chance of cold intolerance.
